# The Effect of Gestational Diabetes Mellitus on the Chorionic Villi of Human Placenta Among Saudi Arabian Mothers: A Quantitative and Comparative Study

**DOI:** 10.7759/cureus.11130

**Published:** 2020-10-24

**Authors:** Abdulrahman Saleh Aldairi, Ahmad Sami A Himayda, Amna Abdul Rahim Moulana, Hasan Sharif H Bukhari, Iyad M Hakeem, Wagih Gamal Eldin Abd E Elbarrany

**Affiliations:** 1 Department of Anatomy, Faculty of Medicine, Umm Al-Qura University, Makkah, SAU; 2 Anatomical Pathology Unit, Department of Laboratory and Blood Bank, Maternity and Children's Hospital, Ministry of Health, Makkah, SAU

**Keywords:** quantitative, chorionic villi, gestational diabetes mellitus, fetus, pregnancy

## Abstract

Background

The placenta is a temporary pivotal organ - the gate between the mother and the fetus. It has multiple functions such as nutrient uptake, elimination of waste products, gas exchange, and production of some vital hormones. However, the pregnancy state is a diabetogenic condition caused by insulin resistance, resulting from physiological variations. Gestational diabetes mellitus (GDM) can have an impact on both the mother and the fetus by causing numerous complications. In our research study, we aim to study and compare the quantitative effect of GDM at the microscopical level within the chorionic villi of the placenta of both mothers diagnosed with GDM and healthy mothers as well as the clinical correlation.

Methods

After applying the inclusion and exclusion criteria, we collected 84 placental samples from February 2017 until May 2017, which were composed of 42 GDM samples and 42 healthy samples. All of these samples have been studied under a light microscope for measuring different parameters.

Results

We found that some of the measured parameters among diabetic villi were lower than those of healthy villi with a p-value < .05 being significant. These include the surface area of the blood vessels (P = .008), the perimeters of the blood vessels (P = .002), the placental barrier thickness/perimeters of the villous blood vessels ratio (P ≤ .001), the placental barrier thickness/surface area of the blood vessels ratio (P ≤ .001), the number of Hofbauer cells/surface area of the villous ratio (P ≤ .001), the number of the blood vessels/surface area of the villous ratio (P = .001), the surface area of the blood vessels/surface area of the villous ratio (P = .004), and the perimeters of the blood vessels/surface area of the villous ratio (P ≤ .001). These parameters have significant effects on fetal development as well as the mother’s status.

Conclusions

GDM is associated with multiple changes in the placenta. Moreover, these changes can impact the fetoplacental circulation and cause multiple complications for the mother and the fetus. Therefore, identifying pregnant women with GDM and controlling hyperglycemia will improve the outcomes of the pregnancy.

## Introduction

The placenta of the human being is considered to be a decisive organ that is responsible for waste eliminations, nutritional uptake, and gas exchanging [1]. Morphologically, it is oval with three surfaces; one side is called the maternal surface, and the other side is called the fetal surface, while a third surface can be considered the peripheral margin which is the outer limit of the vascular plate [2]. On one side, the maternal surface is described as a rough surface (granular) and composed of 15-30 multiple lobes that rise as fissures or grooves [3]. In contrast, the amnion membrane closely covers the fetal surface with the umbilical cord that is attached at almost the central point of the fetal surface of the placenta [3,4]. Furthermore, the placenta is responsible for the production of essential hormones like progesterone and human chorionic gonadotropin, which play an essential role in the maintaining of pregnancy state [1]. The placenta itself is considered a window by which we can understand maternal dysfunctions and their influence on the fetus [3].

At the beginning of the third week of intrauterine life, the placenta starts to accommodate the embryo [2]. Therefore, pregnancy becomes a diabetogenic condition that reflects physiological variations by causing insulin resistance to accommodate the embryo’s demand [5]. Meanwhile, in the healthy physiology of pregnancy, glucose tolerance decreases in the last trimester despite having plasma levels of insulin increase [5].

There are multiple factors for the increase of diabetes mellitus (DM) cases among women of childbearing age. It includes sedentary lifestyle, adolescent obesity, and the virtual epidemic of childhood obesity, with approximately 2% to 5% of all pregnancies affected by diabetes [3]. DM is classified into pregestational diabetes “overt,” which is defined as a female diagnosed with type 1 DM (formerly known as insulin-dependent DM) or type 2 DM (formerly known as non-insulin-dependent DM) while in pregnancy, another classification added is gestational diabetes [3].

Gestational diabetes mellitus (GDM) is the first recognition of any level of glucose intolerance during pregnancy interval [6,7]. In general, a uniform detection and classification of GDM can be done by this definition, but in nonpregnant women who are in their reproductive age, the routine screening of hyperglycemia is not widely performed [7]. According to the American Diabetes Association (ADA) 2020 guidelines, GDM can be defined as the diagnosis in the second or last trimester of pregnancy that was not pregestational diabetes [7]. There have been numerous studies that have described the morphological structure of the placenta and villi with evidence suggesting that strict glycemic control may prevent any major effect and outcomes [8]. However, this area of study has been less frequently explored in the Middle East; nonetheless, Saudi Arabia has only one reported study that was previously done on mothers diagnosed with hypertension [9]. Subsequently, the placenta of women with DM is an exciting area for scientific research studies, and there are multiple pathological changes have been described in the prior literature [10]. In this present study, the aim is to figure out the quantitative effect of GDM on the anatomical level with the pathological level of the chorionic villi of the placenta and the connection between these two levels on the clinical application.

## Materials and methods

This study was approved by the research ethics committee of Umm Al-Qura University, Makkah, Saudi Arabia, as well as Maternity and Children’s Hospital, Makkah, Saudi Arabia. It results from a collaboration between the Department of Anatomy at Umm Al-Qura University and the Anatomical Pathology Unit of the Department of Laboratory and Blood Bank at Maternity and Children’s Hospital in Makkah. It was conducted from February 2017 until May 2017. The study objectives were explained to all patients, and informed consent was obtained. Also, the confidentiality of the information was assured.

A total of 84 placental samples were taken immediately after delivery from the maternal side of the placenta. The samples included 42 healthy samples and 42 diabetic samples (GDM) to be investigated microscopically. Each specimen has been preserved in 10% formalin, prepared, and subjected to sectioning. These sections from formalin-fixed and paraffin-embedded (FFPE) placental tissues with section thickness of three micron. Additionally, the specimens were stained with hematoxylin and eosin for the quality of placental tissue. The images of each specimen were scanned and digitalized using a highly computerized image analysis system of a high-resolution digital camera that is attached to the light microscope and an IBM-compatible computer.

Moreover, these specimens were examined and scanned using light microscope with an original magnification of X400 and the final magnification was X1000. The parameters were done through image analysing system after magnification of the original photos. Both the study group and the control group were examined and measured for different parameters, including the surface area of the villous, the number of the blood vessels, the surface area of the blood vessels, the perimeters of the blood vessels, the number of Hofbauer cells, and the placental barrier thickness. After that, we compared the results of the above parameters, including the number of the blood vessels/surface area of the villous, the surface area of the blood vessels/surface area of the villous, the perimeters of the blood vessels/surface area of the villous, the number of Hofbauer cells/surface area of the villous, the placental barrier thickness/surface area of the blood vessels, and the placental barrier thickness/perimeters of the villous blood vessels.

The inclusion criteria were established as the study group of pregnant Saudi women with confirmed GDM between 24 and 28 gestational weeks, by applying the ADA’s criteria of 100-gram oral glucose tolerance test (OGTT) [7]. OGTT is considered to be abnormal if they had at least two venous plasma glucose values of fasting; ≥ 95 mg/dl (5.3 mmol/dl), one hour; ≥ 180 mg/dl (10.0 mmol/dl), two hours; 155 mg/dl (8.6 mmol/dl), three hours; ≥ 140 mg/dl (7.8 mmol/dl). Babies must have been delivered full term (39 0/7 weeks through 40 6/7 weeks of gestation) [11], be a singleton newborn by either vaginal route or Cesarean section. The control group included pregnant women without any medical issues before pregnancy, plus no history of any complications throughout pregnancy. Additionally, their laboratory test results were all within reference ranges. These patients were included in our research study.

Exclusion criteria consisted of pregnant women who experienced medical conditions before pregnancy or during pregnancy, such as hypertension, pregestational diabetes, hypothyroidism, multiple pregnancies, anemia, cardiovascular disease, respiratory disease, cerebrovascular disease, renal disease, polycystic ovary syndrome, psychiatric illness, and smoking. These individuals were excluded from the present research for both the study group and the control group.

**Figure 1:**
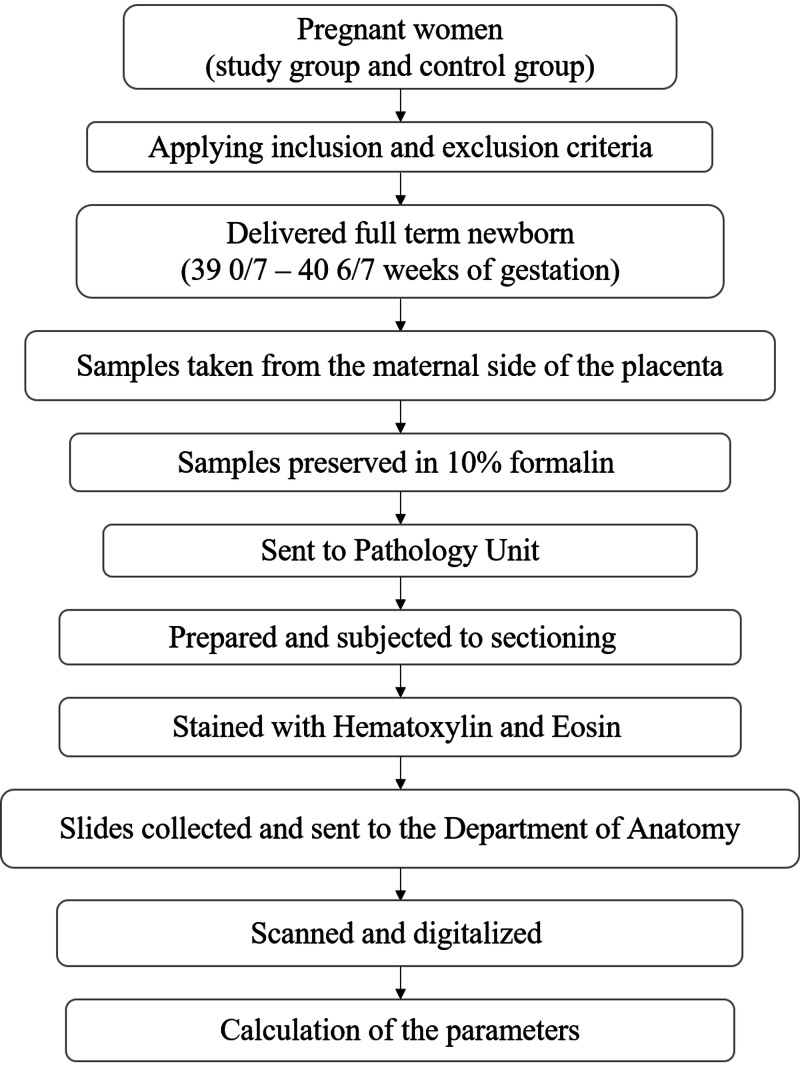
The research workflow

For our statistical analysis, the data were processed and analyzed by using IBM SPSS Statistics for Windows, Version 25.0. (Armonk, NY: IBM Corp.). The results were summarized for each parameter, including numbers and percentages (minimum + maximum + mean + standard deviation (SD) + median score) and compared by performing Mann Whitney U-test. The set of p-value < .05 was statistically significant. Figure 1 presents the algorithm of the workflow of this research article.

## Results

Different parameters were applied for discovering the effect of GDM on the chorionic villi of the placenta by examining it under the light microscope. The results showed greater changes and reductions occurred in the placenta of diabetic (GDM) villi through measuring some parameters within diabetic villi, comparing it with the results of healthy villi (Table 1).

**Table 1:** Description of different parameters within healthy villi Abbreviation: SD, standard deviation.

	Healthy Villi						
Parameter	Min	Max	Mean	SD	Median	25th percentile	75th percentile
The placental barrier thickness (µ)	10.6	809.1	128.9	126.8	101.2	53.7	161.6
The number of Hofbauer cells	0.0	5.0	2.5	1.3	2.0	2.0	3.0
The surface area of the blood vessels (μ^2^)	12895.1	523957.5	107338.0	105474.8	68531.9	30635.1	125385.4
The perimeters of the blood vessels (µ)	441.0	8202.8	1959.2	1780.6	1214.8	842.1	2419.0
The number of the blood vessels	0.0	8.0	3.1	2.1	3.0	2.0	4.0
The surface area of the villous (μ^2^)	195624.2	2395629.3	989101.6	519510.5	836488.7	637191.4	1483223.2
The placental barrier thickness/perimeters of the villous blood vessels ratio	0.721591	53.176290	17.690717	13.242211	13.513306	7.526904	28.527810
The placental barrier thickness/surface area of the blood vessels ratio	0.000650	1.677623	0.273575	0.423930	0.045728	0.013159	0.286780
The number of Hofbauer cells/surface area of the villous ratio	0.000074	0.211817	0.005724	0.033870	0.000284	0.000196	0.000364
The number of the blood vessels/surface area of the villous ratio	0.000001	0.000008	0.000004	0.000002	0.000004	0.000003	0.000004
The surface area of the blood vessels/surface area of the villous ratio	0.015626	0.425395	0.128724	0.111073	0.105644	0.030182	0.190623
The perimeters of the blood vessels/surface area of the villous ratio	0.000000	0.000162	0.000032	0.000036	0.000023	0.000013	0.000039

A description of different findings within healthy and diabetic villi. There was a high reduction in the placental barrier thickness (µ), and hence the min = 7.4, max = 224.4, mean = 95, SD = 58.7, and the median score was 88.1. This reduction was evident at the 25th percentile (49.6) and 75th percentile (128.3) compared to the placental barrier thickness (µ) of healthy villi; min = 10.6, max = 809.1, mean = 128.9, and a large SD of 126.8 was found. The median score was 101.2, the 25th percentile was 53.7, and the 75th percentile was 161.6, higher than that within diabetic villi (Table 2).

**Table 2:** Description of different parameters within villi of patients with gestational diabetes Abbreviation: GDM, gestational diabetes mellitus; SD, standard deviation

	Diabetic (GDM) Villi						
Parameter	Min	Max	Mean	SD	Median	25th percentile	75th percentile
The placental barrier thickness (µ)	7.4	224.4	95.0	58.7	88.1	49.6	128.3
The number of Hofbauer cells	1.0	5.0	2.6	1.1	2.5	2.0	3.0
The surface area of the blood vessels (μ^2^)	11734.6	164600.4	49516.8	40256.5	31913.9	24663.6	63067.3
The perimeters of the blood vessels (µ)	417.7	1777.7	871.5	382.0	739.2	615.7	990.5
The number of the blood vessels	1.0	12.0	2.8	2.3	2.0	1.0	3.0
The surface area of the villous (μ^2^)	202812.6	2755335.7	1034807.5	478874.7	982653.4	703312.1	1257059.7
The placental barrier thickness/perimeters of the villous blood vessels ratio	0.000016	0.000232	0.000094	0.000055	0.000084	0.000054	0.000136
The placental barrier thickness/surface area of the blood vessels ratio	0.000138	0.012986	0.003362	0.002987	0.002163	0.000845	0.005199
The number of Hofbauer cells/surface area of the villous ratio	0.007969	0.392490	0.062962	0.074058	0.033412	0.022665	0.061652
The number of the blood vessels/surface area of the villous ratio	0.000001	0.000007	0.000003	0.000001	0.000002	0.000002	0.000003
The surface area of the blood vessels/surface area of the villous ratio	0.007969	0.392490	0.062962	0.074058	0.033412	0.022665	0.061652
The perimeters of the blood vessels/surface area of the villous ratio	0.000233	0.005736	0.001114	0.001072	0.000767	0.000557	0.001179

The number of Hofbauer cells was similar within diabetic villi, and healthy villi, except SD, which was 1.3 at healthy villi and 1.1 at diabetic villi. The surface area of the blood vessels (μ2) of healthy villi was min = 12895.1, max = 523957.5, mean= 107338, which sharply decreased to min= 11734.6, max= 164600.4, mean= 49516.8 within diabetic villi. The SD and median scores were reduced from 105474.8 and 68531.9 to 40256.5 and 31913.9, respectively. This decrement was also observed at the 25th percentile and 75th percentile. Regarding the perimeters of the blood vessels (µ), a tremendous reduction occurred within diabetic villi: min = 417.7, max= 1777.7, mean= 871.5, and SD = 382, compared to healthy villi with a min = 441, max = 8202.8, mean= 1959.2, and an SD = 1780.6. This reduction was reported at the median score and at both of the 25th percentile and 75th percentile, which diminished from 842.1-2419 to 615.7-990.5, respectively. Concerning the number of the blood vessels, an increment was observed within diabetic villi with a max = 12 compared to a max = eight within healthy villi. Additionally, the surface area of the villous (μ2) was higher within diabetic villi: min= 202812.6, max= 2755335.7, and mean= 1034807.5, compared to healthy villi with min= 195624.2, max= 2395629.3, and mean= 989101.6. The SD was higher within healthy villi (SD = 519510.5) compared to diabetic villi (SD = 478874.7). The median score of the diabetic villi was higher (982653.4) than healthy villi (836488.7). This increment was observed at the 25th percentile, which was 637191.4 within healthy villi and increased to 703312.1 within diabetic villi. There was a decrease at the 75th percentile within diabetic villi compared to healthy villi (1257059.7 and 1483223.2, respectively).

The comparison results of dividing the placental barrier thickness/perimeters of the villous blood vessels, the placental barrier thickness/surface area of the blood vessels, the number of Hofbauer cells/surface area of the villous, the number of the blood vessels/surface area of the villous, the surface area of the blood vessels/surface area of the villous, and the perimeters of the blood vessels/surface area of the villous and within diabetic villi, showed that the results were extreme (zero). In contrast, in healthy controls, the results were higher than that of diabetic villi, especially in the placental barrier thickness/perimeters of the villous blood vessels and the surface area of the blood vessels/surface area of the villous.

By comparison of diabetic and healthy villi through Mann Whitney U-test, a significant difference was observed at the parameters of the surface area of the blood vessels (μ2; P = .008), the placental barrier thickness/perimeters of the villous blood vessels ratio (P ≤ .001), the perimeters of the blood vessels (μ; P = .002), the placental barrier thickness/surface area of the blood vessels ratio (P ≤ .001), the number of Hofbauer cells/surface area of the villous ratio (P ≤ .001), the number of the blood vessels/surface area of the villous ratio (P = .001), the surface area of the blood vessels/surface area of the villous ratio (P = .004), the perimeters of the blood vessels/surface area of the villous ratio (P ≤ .001). There was no significant difference between diabetic and healthy villi in the other parameters.

## Discussion

GDM is a metabolic disease that commonly occurs during pregnancy and can lead to maternal and fetal complications over the short and long-term [12]. The pathophysiology behind GDM is still a matter of much debate as the connection between maternal glycemic control and placental abnormalities remains unclear [1,12]. Placental leptin, an important cytokine that regulates the placental function, is responsible for proliferation, protein synthesis, invasion and apoptosis in placental cells, making it the most probable causative pathophysiological changes in GDM [13]. In the present study, we focused on studying different parameters to see the effect of GDM, considering a p-value < .05 to be significant. We compared diabetic versus healthy villi, and we found a significant decrease in many parameters. As shown in Table 3, there was a decrease at diabetic (GDM) villi comparing to healthy villi, which are the surface area of the blood vessels, the perimeters of the blood vessels, the ratio of the placental barrier thickness to the perimeters of the villous blood vessels, the ratio of the placental barrier thickness to the surface area of the blood vessels, the ratio of the Hofbauer cells to the surface area of the villous, the ratio of the number of the blood vessels to the surface area of the villous, the ratio of the surface area of the blood vessels to the surface area of the villous, and the ratio of the perimeters of the blood vessels to the surface area of the villous.

**Table 3:** Comparison between gestational diabetes villi and healthy villi Abbreviation: GDM: gestational diabetes mellitus. a) All statistical analyses of the parameters have been done by performing Mann Whitney U-test b) Significant at p-value less than .05 c) No significant change

Parameter	GDM (n=42)	Healthy (n=42)	P-Value^a,b^
The placental barrier thickness (µ)	95 ± 58.7	128.9 ± 126.8	.199^c^
The number of Hofbauer cells	2.6 ± 1.1	2.5 ± 1.3	.827^c^
The surface area of the blood vessels (μ2)	49516.8 ± 40256.5	107338 ± 105474.8	.008
The perimeters of the blood vessels (µ)	871.5 ± 382	1959.2 ± 1780.6	.002
The number of the blood vessels	2.8 ± 2.3	3.1 ± 2.1	.162^c^
The surface area of the villous (μ^2^)	1034807.5 ± 478874.7	989101.6 ± 519510.5	.668^c^
The placental barrier thickness/perimeters of the villous blood vessels ratio	0.000094 ± 0.000055	17.690717 ± 13.242211	≤ .001
The placental barrier thickness/surface area of the blood vessels ratio	0.003362 ± 0.002987	0.273575 ± 0.42393	≤ .001
The number of Hofbauer cells/surface area of the villous ratio	0.062962 ± 0.074058	0.005724 ± 0.03387	≤ .001
The number of the blood vessels/surface area of the villous ratio	0.000003 ± 0.000001	0.000004 ± 0.000002	.001
The surface area of the blood vessels/surface area of the villous ratio	0.062962 ± 0.074058	0.128724 ± 0.111073	.004
The perimeters of the blood vessels/surface area of the villous ratio	0.001114 ± 0.001072	0.000032 ± 0.000036	≤ .001

In contrast, there was no significant decrease between diabetic and healthy villi after measuring the other parameters such as placental barrier thickness, the number of Hofbauer cells, the number of the blood vessels, and the surface area of the villous area. The surface area of the villous structures was reported in the literature previously; though this is a controversial point, the explanation could be an adaptive response to the uncontrolled hyperglycemia in which the less glycemic control, the greater the surface area [14]. Also, we can consider this response as an initial sign of adaptation to diabetes-related hypoxia, which might be due to metabolic alterations leading to intrauterine hypoxia [15]. Moreover, a previous study on a hypothetical model reported that the increases in the exchange surface area were noticed in the hyperglycemic state of the mother and the fetus to ensure adequate oxygen delivery to the fetus [8]. Vascular pathological changes were reported in the literature, and most of these changes are detected in the stem villi in different sizes, which end up affecting both arteries and veins [10]. This lesion will affect the fetoplacental circulation [16]. Therefore, the enhancement of the feto-placental angiogenesis by glycemic control occurs purely by longitudinal growth [17].

On the other hand, multiple research studies of GDM established that hyperglycemia appeared to participate in the morphometric abnormalities [8]. Furthermore, uncontrolled GDM could contribute to increasing fibrin in the placenta and the development of villous edema [18]. Hence, good glycemic control during pregnancy could reduce the clinical complications related to the maternal and fetal side [19]. Even after controlling the blood glucose levels to become euglycemia, the pathological features associated with GDM are irreversible, but the clinical pregnancy outcomes will be improved [20,21].

In our study, and based on this previous information, we do not know how much the damage to the applied parameters was because of the unknown degree of insulin used or glycemic control [22]. Accordingly, there are some limitations in our research study, such as insulin use and glycemic control. We recommend that the early screening of hyperglycemia be related to decreased maternal and fetal complications. This will improve fetoplacental circulation, especially in pregnant women with high-risk factors such as marked obesity, glycosuria, a strong family history of DM [23], and according to the ADA, most cases of hyperglycemia represent pregestational diabetes, especially in those with high-risk factors. Based on this, we suggest the early screening of DM as much as possible in those with the above risk factors [7]. However, further research is needed to cover this vast topic because there are more parameters to be studied and other factors such as smoking, alcohol use, morbid obesity, insulin use with GDM, type 1 DM, and type 2 DM.

## Conclusions

From an anatomical and pathological point of view, we identified a significant decrease in some parameters evaluated between the placenta of mothers diagnosed with GDM and the placenta of healthy mothers. Moreover, these pathological changes will affect the fetoplacental circulation. Therefore, identifying the cause of glucose intolerance is extremely important, particularly in those with high-risk factors. Testing must be done as soon as possible because not all cases of hyperglycemia will represent GDM. By this, we can decrease multiple complications and improve the outcomes for the mother and the fetus.
